# A Novel Concept of Nano-Enhanced Phase Change Material

**DOI:** 10.3390/ma17174268

**Published:** 2024-08-29

**Authors:** Răzvan Calotă, Octavian Pop, Florin Bode, Cristiana Croitoru, Andrada Serafim, Alina Bărbulescu, Celina Damian, Lucia Tefas

**Affiliations:** 1 CAMBI Research Centre, Technical University of Civil Engineering Bucharest, 66 Pache Protopopescu Bd., 021414 Bucharest, Romania; razvan.calota@utcb.ro (R.C.); cristiana.croitoru@utcb.ro (C.C.); 2 Faculty of Building Services, Technical University of Cluj-Napoca, 103–105 Muncii Bd., 400461 Cluj-Napoca, Romania; octavian.pop@termo.utcluj.ro; 3 AtFlow Research Centre, Technical University of Cluj-Napoca, 103–105. Muncii Bd., 400461 Cluj-Napoca, Romania; 4 Advanced Polymer Materials Group, National University of Science and Technology Politehnica Bucharest, 313 Splaiul Independentei, 060042 Bucharest, Romania; andrada.serafim0810@upb.ro (A.S.); celina.damian@upb.ro (C.D.); 5 Department of Civil Engineering, Transilvania University of Brașov, 5 Turnului Str., 500152 Braşov, Romania; 6 Faculty of Pharmacy, luliu Hațieganu University of Medicine and Pharmacy, 8 Victor Babeș Str., 400102 Cluj-Napoca, Romania; tefas.lucia@umfcluj.ro

**Keywords:** NEPCMs graphene oxide, metallic nanopowder, PCMS

## Abstract

In the actual context of growing concerns over sustainability and energy efficiency, Phase Change Materials (PCMs) have gained attention as promising solutions for enhancing energy storage and release efficiency. On another hand, materials based on graphene oxide (GO) have proven antibacterial activity, biocompatibility, efficiency in microbial growth inhibition, and pollutant removal. Integrating nanoparticles into PCMs and creating Nano-Enhanced Phase Change Materials (NEPCMs) have opened new horizons for optimizing the performance of these systems and sustainable development. The key objective of this work is to gain insight into NECPMs, which are used in solar wall systems to enhance solar energy storage. Paraffin RT31 was mixed with Cu nanoparticles, graphene oxide (GO), and Cu-decorated GO (Cu@GO) at loading ratios ranging from 1% to 4% (*w*/*w* nanoparticles with respect to RT31). The compositions were characterized through Differential Scanning Calorimetry (DSC) and rheology tests. The decoration of the carbon-based nanoparticles was performed using the ultrasonication procedure, and the decoration efficiency was confirmed through X-ray Photoelectron Spectroscopy (XPS). The rheologic measurements were performed to correlate the flow behavior of the NEPCM with their composition at various temperatures. The study methodically investigated these composites’ latent heat values, phase change peak temperatures, and solidification phase change temperatures. Compared to pure paraffin, the solidification of the formulations obtained using Cu@GO exhibits the largest increase in latent heat, with a 12.07% growth at a concentration of 2%. Additionally, at a 4% concentration of NEPCM, the largest increase in thermal conductivity was attained, namely 12.5%.

## 1. Introduction

The current technology trends align with the worldwide movement towards reducing energy consumption and sustainable development using low-environmental impact materials. The building industry is one of the most energy-intensive areas where urgent measures are needed, being one of the biggest energy consumers responsible for more than 45% of the global energy demand [1]. Generally, actions that can be employed in the construction sector to improve energy efficiency, increase sustainability, and minimize carbon footprint include using solar energy, energy storage, and incorporating significant thermal insulation. Improved thermal energy storage through Phase Change Materials (PCMs) has become popular because these materials can store significant thermal energy via phase changes. Techniques for improving heat-transfer properties, including encapsulation, expanded surfaces, and conductive particle dispersion, have been investigated [2]. Therefore, employing advanced technologies and materials such as nano-particle-loaded PCMs enhances the effectiveness of harnessing solar energy. Several research studies have shown that using fins or nanoparticles can enhance the performance of PCMs in heat transfer and improve their thermal efficiency [3,4,5]. In the context of solar air collectors, incorporating Nano-Enhanced PCMs (NEPCM) during daylight hours allows for the storage of solar heat energy. Placing these NEPCMs within the solar air heater elevates the surrounding air temperature as it passes through the collector. This warm air can be used in air conditioning during the cold season [6]. Hot air prepared in collectors integrating NEPCM can be utilized for heating, fruit and vegetable drying, dairy production, and sun cooking [7].

Other studies provide insights regarding the performance of various materials installed within solar collectors or other construction-related systems. Carbon-based nanoparticles, such as graphene, graphite, graphene nanoplatelets, or various metals and their oxides, are among the materials used to enhance the PCM’s characteristics [8,9]. Metal addition provides good thermal conductivity, although thermal conductivity at high temperatures varies due to suppression induced by PCM density change [10]. At the nanometric scale, current nanotechnology enables the production of even more materials of various types [11]. In a recent study [12], ZnO was dispersed in paraffin wax in a weight ratio between 0.5% and 3%. The results indicated that the addition of 2.5 wt.% ZnO leads to the highest increase in thermal performance (18.84%) and heating and cooling cycles (34.4%). Anandan et al. [13] showed that adding 1% CuO leads to a 52% increase in solid-state thermal conductivity and a 20% increase in a liquid state. In addition, the solidification and melting period decreases with increasing CuO ratio by 18.33% and 16.6%, respectively. Venkateshwar et al. [14] investigated the impact of nanoparticle size and concentration on PCM stability, which directly impacts the overall performance of NEPCMs. The authors utilized two different concentrations of CuO (50 nm) and iron oxide (50–100 nm) (0.5% and 1.0 wt.%). They found that the size of the nanoparticles has a higher influence on the compositional properties than their density. Furthermore, the concentration of nano species is crucial, as increasing their concentration increases the mixture’s viscosity [14]. 

Although many metals or their oxides have been investigated for NEPCM, Cu remains one of the favorites due to its remarkable thermal properties [15,16,17]. Lin et al. [18] showed that adding 2% weight of Cu in paraffin wax leads to an increase of the thermal conductivity of over 45%, while Zhu et al. [19] proved that adding CuO nanowires leads to improvement of the thermal conductivity far greater than the nanoparticles.

Carbon-based materials, especially in their nano-form, have also been exploited for applications in thermal energy storage due to their extraordinary thermal conductivity. For example, carbon nanotubes exhibit a thermal conductivity of more than 2000 W/mK [20], over five times higher than copper. The addition of graphene in a loading ratio of only 2% in paraffin led to an increase of 60% in thermal conductivity [20].

NEPCMs are commonly prepared using the sol-gel technique, interface polymerization, emulsion polymerization, and in situ polymerization methods. However, the presence of supercooling throughout the solidification process results in inadequate solidification, particularly in salt hydrate and other composite materials. As a result, the heating and cooling operations of the NEPCM must be thoroughly considered.

The present article aims to assess the potential of NEPCMs obtained through the dispersion in paraffin (RT31) of various particles: copper oxide (CuO), graphene oxide (GO), and copper-decorated graphene oxide (Cu@GO). The article explores the synergistic effect of Cu and GO on the latent heat values and phase change temperature by employing Cu@GO. Various loading ratios between 1% and 4% weight (nanoparticles: paraffin) were used, and the compositions of the three formulations were correlated with their thermal and rheologic characteristics.

## 2. Materials and Methods

### 2.1. Materials

R31 (RubiTherm, Berlin, Germany) was selected as the PCM model. CuO (Aldrich, Merck, Darmstadt, Germany) 50 nm, according to manufacturer’s datasheet) and GO (Sigma Aldrich, Merck, Darmstadt, Germany) 15–20 sheets, according to manufacturer’s datasheet) were employed to obtain NEPCM. CuSO_4_ (Merk) was used to decorate the GO nanopowder. 

### 2.2. Synthesis of the Composite PCM

#### 2.2.1. Decoration of Graphene Oxide (GO)

GO was decorated with Cu through an ultrasonical procedure, previously described in the literature [21,22]. To this end, a dispersion of 1% GO (*w*/*vol*) was prepared in a 10% (*w*/*vol*) CuSO_4_ aqueous solution. The prepared dispersion was maintained under sonication for 60 min. A VCX 750 ultrasonic device (Sonics & Materials, Inc., Newton, CT, USA) equipped with a Ti-6Al-4V probe tip and a 750 W processor was employed. The apparatus was operated at 20 kHz, and the vibration amplitude of the probe was set to 30%. Following ultrasonication, the dispersion was centrifuged using a Hettich Universal 320 centrifuge (Hettich, Beverly, MA, USA) at 2500 rpm for 5 min. The nanoparticles were washed through re-dispersion in water until the aquilot was colorless. Then, it was filtered, dried, and stored at room temperature until further use.

#### 2.2.2. NEPCM Synthesis

The paraffin was weighed and placed in a Berzelius beaker, and the appropriate amount of nanopowder was added to achieve the pre-established nanopowder: paraffin ratio, as detailed in Table 1.

The dispersion was subject to ultrasonication for one hour. A VCX 750 ultrasonic device from Sonics & Materials, Inc. (Newton, CT, USA) equipped with a Ti-6Al-4V probe tip and a 750 W processor operating at 20 kHz was used for this purpose. The vibration amplitude of the probe tip was set to 30%. Following synthesis, NEPCMs were stored in polypropylene tubes at room temperature for further characterization. 

The formulations and the nanopowder loading ratio are also presented in Table 1.

### 2.3. Characterization of the Obtained Materials

#### 2.3.1. Evaluation of the Decoration’s Efficiency

The decoration efficiency of GO nanostructures was estimated through an X-ray Photoelectron Spectroscopy (XPS) using a K-Alpha instrument from Thermo Scientific (Thermo Fischer Scientific, Waltham, MA, USA), bearing a monochromatic AlKα source (1486.6 eV), at a bass pressure of 2 × 10^−9^ bar. The pass energy for survey spectra was 200 eV, while the C1’s high-resolution spectra were registered using a pass energy of 20 eV. Deconvolution of high-resolution C1s spectra was performed by applying a Gaussian–Lorentzian convolved algorithm.

#### 2.3.2. Thermal Conductivity Evaluation

The method introduced in [23] was utilized to evaluate the thermal conductivity, $\lambda_{NEPCM},$ of Cu@GO at different concentrations in the mixture.

(1) $$\lambda_{NEPCM}=\frac{\lambda_{nano}+2\lambda_{PCM}-2\varphi \left(\lambda_{PCM}-\lambda_{nano}\right)}{\lambda_{nano}+2\lambda_{PCM}+\varphi \left(\lambda_{PCM}-\lambda_{nano}\right)}\times \lambda_{PCM}+\;\frac{4.22035\times 10^{5}}{{\left(100\times \varphi \right)}^{-1.07304}}\times \varphi \times \rho_{PCM}\times c_{p,\;PCM}\times \sqrt{\frac{\kappa \times T}{\rho_{nano}\times d_{nano}}\times }f\left(T,\varphi \right)$$

where:

(2) $$\;f\left(T,\varphi \right)=\frac{\left(2.8217\times 10^{-2}\varphi +3.917\times 10^{-3}\right)T}{T_{0}}-3.0699\times 10^{-2}\times \varphi -3.91123\times 10^{-3}$$

φ—mass fraction of nanomaterial [−];

$\lambda_{nano},\;\lambda_{PCM}$—thermal conductivity of the nanomaterial and PCM, respectively [W/m·K],

$\rho_{PCM}=880\;kg/m^{3}$, $c_{p,PCM}=2\;kJ/kgK$, $d_{nano}=50\times 10^{-9}\;m,$

κ= Boltzmann constant = 1.380649 × 10^−23^ m^2^ kg s^−2^ K^−1^.

The values of thermal conductivity for the Cu@GO nanopowder were chosen according to the technical sheets of the corresponding substance and relevant studies from the literature [24,25,26].

#### 2.3.3. Evaluation of the Flow Behavior of the Synthesized NEPCM

Rheologic measurements were performed using a Kinexus Pro Rheometer (Malvern Instruments Limited, Malvern, Worcestershire, UK). To this end, a plate–plate disposable geometry was used, with an upper diameter of 25 mL. Also, 0.27 mL of each composition was placed on the lower plate of the equipment, and a gap of 0.5 mm was set between the two plates. The measurements were performed at 10 °C (below the melting temperature of the formulations), 30 °C (near the melting point of R31), and 70 °C (above the melting point of the compositions). The flow curves were measured in the 1–100 Hz shear rate interval at a constant strain of 1%.

#### 2.3.4. Assessment of the Thermophysical Properties of the Synthesized NEPCM

Differential scanning calorimetry (DSC) was used to evaluate the thermophysical properties of pure RT31 and nanocomposite formulations. A DSC 822 (Mettler Toledo, Columbus, OH, USA) was used to characterize the samples. The equipment was meticulously calibrated with indium (melting temperature 156.6 °C; specific melting enthalpy 28.54 J/g) and zinc (melting temperature 419.6 °C), ensuring the accuracy of the measurements. The dynamic method, airflow 25 mL/min, and a scan temperature range of 8–50 °C were used for the measurements. The data sheet of the pure paraffin was employed to define the measurement cycles, further ensuring the thoroughness of the experiment. A cooling and heating rate of 1 K/min [27] was used to conduct the experiments. The samples were weighed in sealed 40 μL aluminum crucibles.

## 3. Results 

### 3.1. Decoration Efficiency Assessment

**Figure 1:**
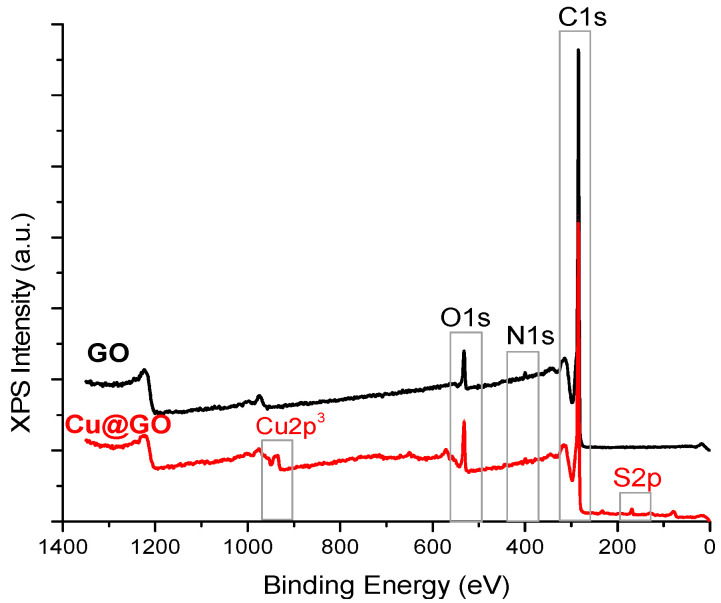
XPS survey spectra for the raw and Cu@GO nanostructures.

The survey spectra shown in Figure 1 and the data from Table 2 shows the surface elemental composition of the GO starting materials and functionalized nanostructures.

The XPS spectra reveal the main peaks of C and O from graphene oxide. At the same time, an apparent modification along with the attachment of the Cu particles on the surface is provided by the presence of Cu2p3 and, respectively, S2p species, similar to the results obtained by Samuel et al. [28].

The success of the decoration can also be assessed by looking at the lower C atomic composition for Cu@GO and increased O atomic ratio, an indication of the surface structure rearrangement obtained after the GO sheets decoration with Cu nanoparticles.

**Figure 2:**
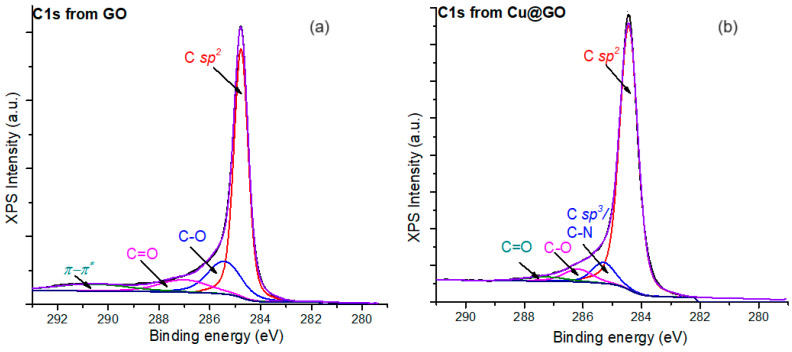
(**a**) High-resolution C1s spectra for the raw; (**b**) functionalized GO nanostructures.

For a more detailed vision of the structure of the raw and functionalized GO nanostructures, we employed deconvolution to obtain the secondary peaks corresponding to C1s electrons, as shown in Figure 2. This method played a crucial role in our research, providing a deeper understanding of the nanostructures.

Thus, four types of C1s species were found for GO nanosheets as follows: the peak at 284.8 eV assigned to C *sp^2^* type species, the peak at 285.5 eV coming from C-O species from the functional groups of GO, then the peak at 287 eV attributed to C=O species from the carboxyl groups of GO, and the peak at 290.6 eV attributed to the π-π* electronic transitions from the aromatic graphitic layers of GO. Furthermore, in the C1s spectra registered for the Cu@GO surface, one can observe a different configuration of the secondary peaks. The peak from 285.3 eV is assigned to C *sp*^3^ or C-N species, while the C-O is shifted to 286.2 eV. At the same time, the C=O peak is stated at 287.3 eV, but the π-π* electronic transitions are disrupted by the presence of the conductive Cu species, in concordance with the findings from other studies [29].

### 3.2. Thermal Conductivity Evaluation

Cu nanoparticles, in conjunction with GO, impacted thermal properties. The large surface area of GO can accommodate more copper nanoparticles, which further improves the thermal properties of the composite by enhancing the heat-conduction pathways within the material. This synergistic effect between the high surface area of GO and the thermal conductivity of copper nanoparticles results in a higher latent heat-storage capacity for the Cu@GO nanocomposite compared to other configurations without such a combination.

**Figure 3:**
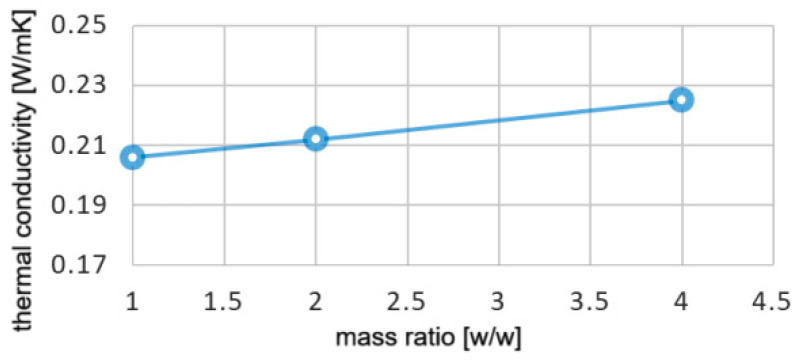
Thermal conductivity for Cu@GO NEPCM.

When compared to simple GO, the Cu@GO nanopowder has roughly 5% higher thermal conductivity, 8% higher density, and nearly 4% lower specific heat. However, when nanomaterials are added to paraffin in low quantities, the respective variations are diluted, resulting in changes in thermal conductivity values only to the third decimal. The variation of thermal conductivity for NEPCM Cu@GO is presented in Figure 3.

### 3.3. Rheologic Behaviour of the NEPCM

**Figure 4:**
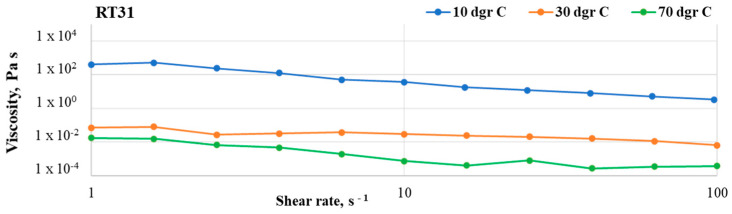
Flow curves registered in the shear rate range of 1–100 s^−1^ for the control sample (RT31) at 10 °C, 30 °C, and 70 °C.

The control sample (Figure 4) exhibited a relatively low dependency of viscosity on shear rate, especially around the melting point.

At 10 °C, the sample is solid and shows some dependency on the shear rate, probably due to the friction between the metal plates. At 30 °C and 70 °C, RT31 shows Newtonian behavior at shear rates above 10 s^−1^, with a viscosity value of around 1.82 × 10^−2^ and 5 × 10^−4^ Pa·s, respectively. The slight viscosity variations are attributed to the slippage between the rheometer plates rather than a dependency on the shear rate. Also, it can be noticed that the differences between the values registered at 30 °C and 70 °C, respectively, are relatively small. This behavior is attributed to the complete melting at 70 °C. However, a decrease of the viscosity at low shear rates may be observed at this temperature.

Small variations of viscosity with the increase of the shear rate can be observed for all composite formulations. A key finding is that regardless of the type or quantity of nanoparticles added to the systems, all materials maintained the viscosity-temperature dependency, showing a consistent decrease of viscosity with the temperature increase.

**Figure 5:**
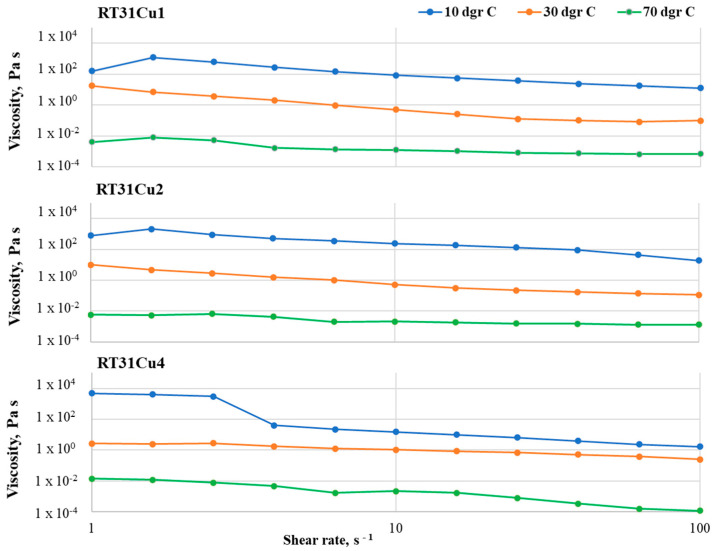
Flow curves registered for the RT31Cu samples with various loading ratios, at 10 °C, 30 °C, and 70 °C, in the shear rate interval 1–100 s^−1^.

At 10 °C, RT31Cu 1 samples show a viscosity drop of one order of magnitude (from 158 to 12 in the case of RT31Cu 1 and 781 to 19 in the case of RT31Cu 2, respectively) at loadings of 1 and 2% (*w*/*w* nanopowder with respect to the RT31) and a viscosity decrease of three orders of magnitude for a CuO loading of 4% (Figure 5).

The viscosity drop is registered at a shear rate of approximately 4 s^−1^ and might be attributed to the rearrangement of the nanoparticles in the composite system. The Newtonian behavior exhibited by the control sample at 30 °C is not maintained in the composite formulations, with RT31Cu compositions having a decrease of viscosity of one (in the case of RT31Cu 4) up to three (in the case of RT31Cu 1) orders of magnitude. It can be observed that the increase of nanopowder amounts leads to a decrease in the viscosity drop with the shear rates around the melting point. At 70 °C, the compositions RT31Cu 1 and RT31Cu 2 exhibit a viscosity drop of one order of magnitude with the increase of the shear rate, while RT31Cu 4 shows a decrease of the viscosity of over 10^−2^ Pa·s.

**Figure 6:**
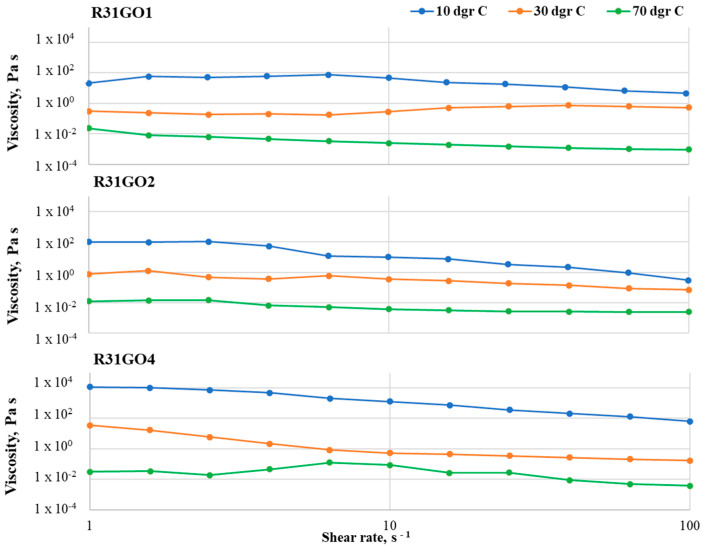
Flow curves registered for the RT31GO samples at 10 °C, 30 °C, and 70 °C.

**Figure 7:**
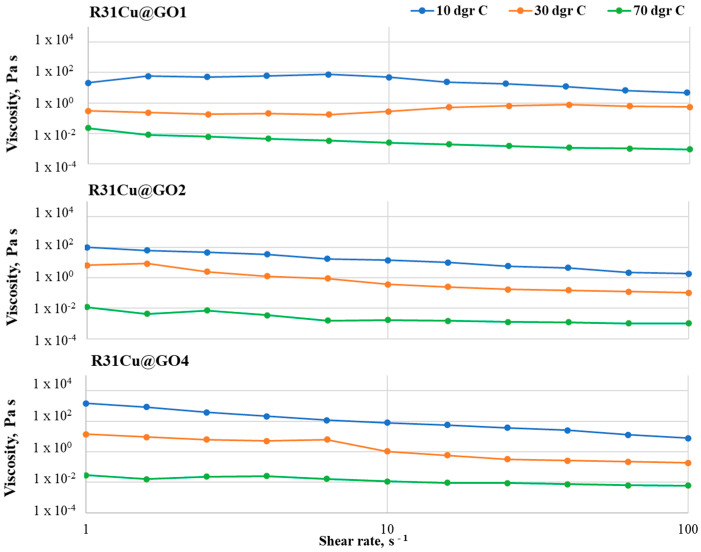
Flow curves registered in the shear rate interval 1–100 s^−1^ for the RT31Cu@GO compositions.

The same shear rate and temperature dependency can be observed in the other two composite systems (Figure 6 and Figure 7). Similarly to RT31Cu, adding nanopowders leads to a non-Newtonian behavior for all studied temperatures and loading ratios. However, the viscosity variations are smaller, especially at low loading ratios. For example, the RT31GO 1 formulation has almost a Newtonian behavior at 30 °C, with a slight increase of the viscosity value at shear rates of over 10 s^−1^. The same compositions have a variation of only an order of magnitude for the other two tested temperatures (10 °C and 70 °C). A slight viscosity increase can also be observed at the sample RT31GO 4 at 70 °C, in the shear-rate interval 2–40 s^−1^. These increases can be attributed to rearranging the nanopowders inside the paraffin matrix during the tests. Noticeable is also a higher decrease of the viscosity (of three orders of magnitude) for the samples RT31GO 2 at 10 °C and 30 °C and sample RT31GO 4 at 10 °C. This behavior is especially interesting at 30 °C because, at this temperature, the control sample (RT31) presents a Newtonian flow, indicating that the nanopowders strongly impact the rheological characteristics of the compositions.

The slightest viscosity modifications with the shear rate can be observed for the sample RT31Cu@GO, especially for the compositions with a loading ratio of 1 and 2% (*w*/*w*) nanopowder with respect to the paraffin. The highest viscosity decrease is registered for RT31Cu@GO 1 at 70 °C and for the composition RT31Cu@GO at 10 °C and 30 °C. At 30 °C, the formulation RT31Cu@GO maintains the Newtonian behavior displayed by the control sample at the same temperature. The sample RT31Cu@GO 2 exhibits a decrease of viscosity of two orders of magnitude for all tested temperatures. Furthermore, the loading ratio seems to influence the compositions’ viscosity only at low temperatures, at 30 °C and, especially at 10 °C.

At 70 °C, the behavior of the composite formulations is mainly determined by the organic matrix, and the presence of nanopowders up to a 4% loading ratio has no significant influence on their flow.

Generally speaking, the viscosity measurements were performed to provide information regarding (1) the ease of manipulating the formulations during the fabrication process and (2) the stability of the formulations. As depicted in Figure 5, Figure 6 and Figure 7, all composite formulations present a slight decrease of the viscosity at shear rates of above 10 s^−1^, which may lead to an ease of the fabrication process. Furthermore, the lack of noise in the registered flow curves indicates that all nanocomposite formulations are stable.

The lack of studies concerned with the rheological transition mechanism from non-Newtonian to Newtonian behavior for nano-particle-based dispersions was discussed in [30]. To this end, Zhuang et al. [30,31] conducted a thorough study using nanofluids based on paraffin and various loadings of CuO, Al_2_O_3,_ and TiO_2_ mass loadings. Their study revealed that the critical shear rate depends on the nanoparticles’ type and loading ratio and also on the temperature at which the measurement is performed. Furthermore, they concluded that the nanofluids’ transition from non-Newtonian to Newtonian behavior as the shear rate increases is due to a de-agglomeration of the compositions. In agreement with their findings, small differences in the critical shear rate can be noticed in compositions R31Cu, R31GO, and R31Cu@GO. For example, while the critical shear rate for R31Cu1 at 30 °C is 25 s^−1^, at the same temperature, but at a 4–fold loading ratio, the critical shear rate is 4 s^−1^. Moreover, as depicted in Figure 4, Figure 5 and Figure 6, a transition from non-Newtonian to Newtonian flow also occurs. Since the critical shear rate is most obvious at a 2% loading, at 70 °C, the change of flow behavior can also be attributed to an improved distribution of the nanoparticles in the organic matrix. At lower temperatures, the organic matrix is solid, and, therefore, the nanoparticles cannot move, while at higher loading ratios, it is expected that the nanospecies agglomerates are harder to break.

### 3.4. Sensibility Study Regarding the Heating and Cooling of the Mixture

**Figure 8:**
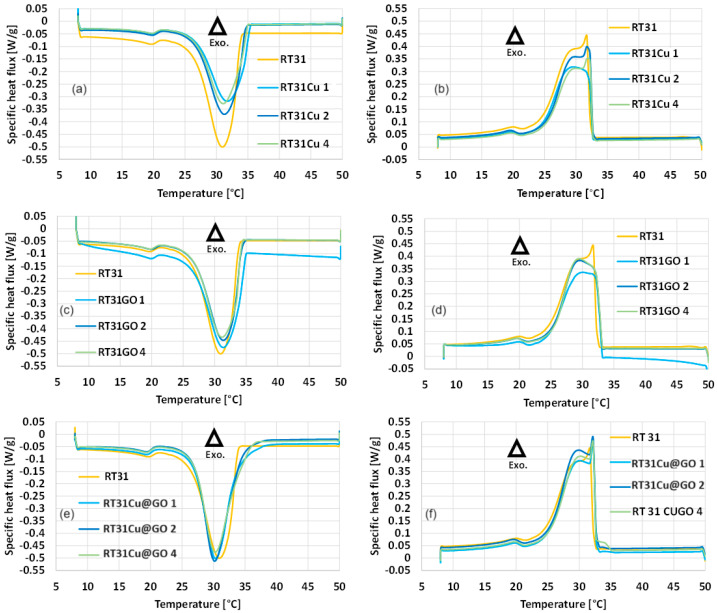
The DSC generated thermograms to evaluate the heat flux variation during phase change. The positive values from the experimental results indicate the exothermic process (∆ Exo.), cooling and solidification. Representation of the specific heat flux (Q_p_) for (**a**,**b**) RT31 with Cu heating and cooling; (**c**,**d**) RT31 with GO heating and cooling; (**e**,**f**) RT31, RT31 with Cu@GO heating and cooling.

The DSC experiments revealed the variation in the thermophysical properties of the studied samples, depending on the type of nanoparticles and concentration size. Figure 8 presents each sample’s specific heat flux (Q_p_) variation during the DSC heating and cooling procedures.

The thermograms indicate the presence of two peaks during both heating and cooling for pure RT31 and the composite samples. The lower peaks indicate changes in the paraffin’s crystalline structure, revealing that the samples undergo a solid-to-solid phase transition at the corresponding temperatures. The curves with the higher peaks that can be identified around the nominal phase change temperature of the pure material are the result of the significant amount of thermal energy exchange during the melting and solidification processes.

Table 3 presents the thermophysical properties of all samples, resulting from the DSC experiment at a heating and cooling rate of 1 K/min for initial temperature (T_on_), peak temperature (T_p_), final temperature (T_end_), specific heat flux (Q_p_), and latent heat (L).

### 3.5. Impact of the Type and Percentage of Nanoparticles on the Thermal Storage Capacity of Paraffin

**Figure 9:**
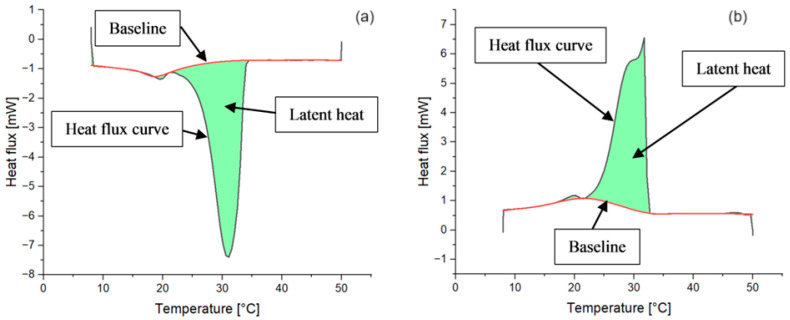
Example of latent heat determination for pure RT31. (**a**) Latent heat determination for RT31 heating; (**b**) latent heat determination for RT31 cooling.

The surface area between the DSC curve and the baseline represents the latent heat values. The OriginPRO software (OriginLab corp. in Northampton, MA, USA) has been employed to perform a baseline correction of each curve and determine the integral values. Figure 9 shows an example of latent heat determination in the case of RT31.

**Figure 10:**
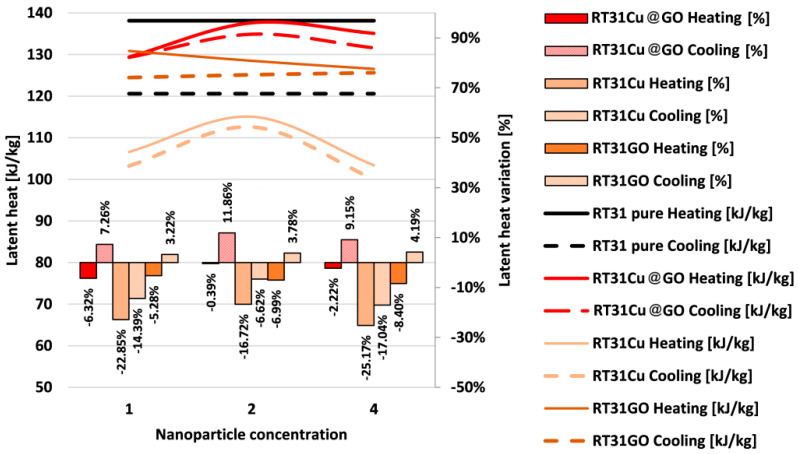
The latent heat curves of the samples and the variations from the pure material.

Figure 10 presents the variation of measured latent heat depending on the type and concentration of nanoparticles compared to the pure material during solidification and melting. The latent heat variation determined by the nanoparticles in comparison to the pure paraffin is also provided and expressed in percentage for clarity.

The latent heat curves for all samples follow similar trends in both melting and solidification. The measured values of the latent heat are generally higher during melting. 

As depicted in Figure 10, during the solidification process, slight increases of about 3.22% in the latent heat values can be noticed when comparing the RT31GO values with those of the pure paraffin. For the melting process, decreases in latent heat are slightly higher, at 5.28%, 6.99%, and 8.40%, for loading ratios of 1%, 2%, and 4%, respectively.

In the case of Cu-loaded nanocomposites, all values are lower than the latent heat of the pure material. In the case of melting, the minimum and maximum decreases, in comparison to the latent heat of the pure material, are observed at RT31Cu 2 with a reduction of 16.72% and at RT31Cu 4 with a reduction of 25.17%, respectively.

For solidification, extreme variations are also observed at RT31Cu 2 and RT31Cu 4. The minimum decrease corresponds to RT31Cu 2, 6.62%, while the maximum is identified at RT31Cu 4, 17.04%.

In the solidification of Cu@GO-loaded RT31, the highest increase in latent heat, compared with pure paraffin, is observed at a concentration value of 2%, yielding an increase of 11.86%. The minimum increase corresponds to the concentration of 1% at an increase of 7.26%.

In the case of melting, minor decreases were observed, ranging from 0.39% to 6.23% in comparison to the pure paraffin.

### 3.6. Impact of the Type and Quantity of Nanoparticles on the Phase Peak Change Temperature

**Figure 11:**
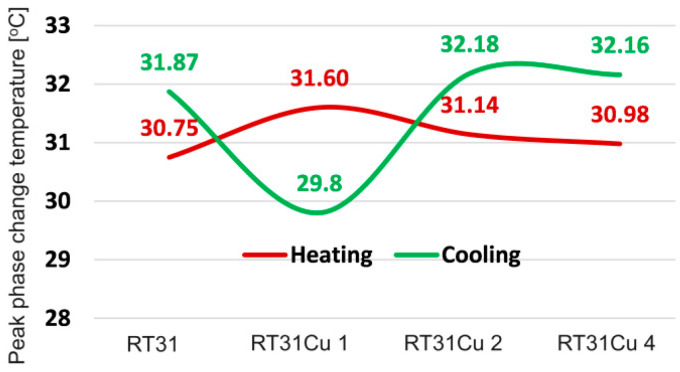
The variation of peak phase change temperatures for the RT31/Cu composition.

The DSC results indicated changes in the peak phase change temperature, the temperature value corresponding to the maximum heat flux. The addition of Cu caused more notable fluctuations. Figure 11 shows Cu’s impact on the peak phase change temperature. The maximum difference of 1.80 °C can be observed for the RT31Cu 1 composition.

**Figure 12:**
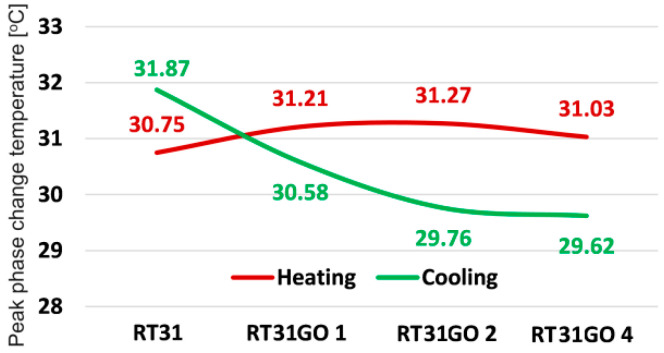
The variation of peak phase change temperatures for the RT31GO composition.

The addition of GO at loading rations of 1, 2, and 4% (Figure 12) immediately reverses the solidification and melting peak phase change temperature order. Solidification occurs at lower peak temperatures, with differences between them in the range of (0.63–1.51) °C.

In the case of cooling, the peak temperatures are higher than in the case of heating.

**Figure 13:**
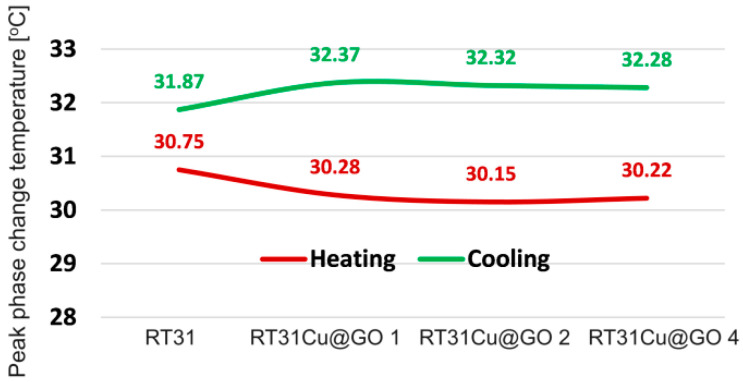
The variation of peak phase change temperatures for the RT31CuGO mixture.

Adding Cu@GO (Figure 13) does not significantly impact the peak phase change temperature values. The variation of peak phase change temperatures with the nanoparticle concentration is relatively constant. The differences between the solidification and melting peak temperatures range from 2.06 °C to 2.17 °C.

Therefore, we can conclude that adding Cu@GO nanoparticles to the phase change material (PCM) affects the phase change peak temperature, leading to a reversal trend where the peak temperature either increases or decreases depending on the concentration and distribution of the nanoparticles.

This behavior can be explained by the enhanced thermal conductivity and uniform distribution of heat within the PCM matrix due to the presence of the Cu@GO nanoparticles. This improved thermal conductivity facilitates a more uniform temperature distribution during the phase change process, which can shift the phase change temperature depending on the specific interactions between the nanoparticles and the PCM.

## 4. Discussion

The present work investigates NEPCM formulations containing three different nanopowders: metallic (Cu), carbon-based (GO), and metal-decorated carbon-based particles (Cu@GO) dispersed in an organic matrix. The influence of the amount and type of nanoparticles has been evaluated from a rheological and thermal point of view.

In agreement with the finding of Zhuang et al. [30], adding nanoparticles leads to compositions with complex rheological behavior. Considering that the particles and organic matrix are free of reactive functional groups, the quantity of nano species has a stronger influence on the synthesized materials’ characteristics than their type, as demonstrated by the results presented in previous sections. Furthermore, all compositions have a shear thinning behavior more pronounced in the low shear rates range, attributed to the disintegration of the nanoparticles’ agglomerates [31].

Bharathiraja et al. [32] added Multi-Walled Carbon Nanotubes (MWCNTs) and nano SiO_2_ to a pure paraffin material with a phase change temperature range of (58–60) °C. 

Four combinations were investigated, differing in nanoparticle concentration amount and mixture configuration: 0.5% MWCNT, 0.5% SiO_2_, 0.5% MWCNT + 0.5% SiO_2_, and 1% MWCNT + 1% SiO_2_. By increasing the nanoparticles’ quantity, a decrease in melting temperature was observed from 62.7 °C, corresponding to the pure material, to 59.7 °C, a value corresponding to the mixture of paraffin + 1% MWCNT + 1% SiO_2_. The peak temperature increased during solidification.

When employing GO, the solidification peak temperatures decrease below the melting temperature values and provide near-mirrored fluctuations.

In [30], the melting latent heat gradually decreased from 189 kJ/kg (pure material) to 176.83 kJ/kg (paraffin + 1% MWCNT + 1% SiO_2_). The solidification latent heat also drops when 1% MWCNT +1% SiO_2_ is added to the pure material; otherwise, it fluctuates slightly between concentrations of 0.5% MWCNT, 0.5% SiO_2_, and 0.5% MWCNT + 0.5% SiO_2_.

Elarem et al. [33] also studied the inclusion of Al and Cu using a base material of pure paraffin characterized by a phase change temperature range of (56–60) °C. The studied materials were mainly paraffin with Al and paraffin with Cu. The nanoparticles were added in concentrations of 0.1, 0.3, 0.6, 1, 2.5, and 5% for each of the two nanoparticles. They noted an inverse variation of the solidification temperatures characterized by a slight drop in phase change temperature and then an increase as the concentrations of Al were higher. When Cu is employed, both increases and decreases of the peak phase change temperatures were observed, similar to most fluctuations emphasized in this study. However, the melting temperature always remained higher than in the case of solidification. The latent heat variations in [33] peaked between concentrations of (0.6–1)% for Cu and dropped as the concentration increased.

**Figure 14:**
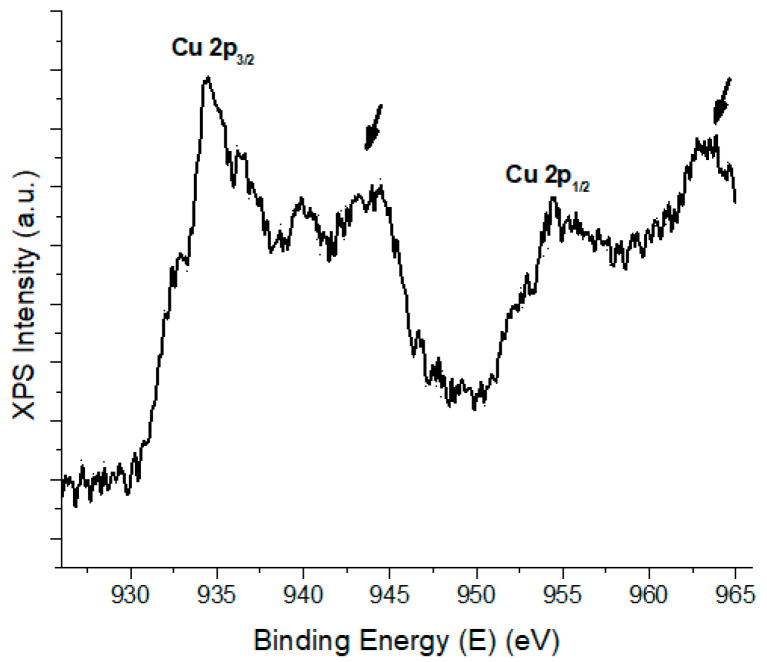
XPS Intensity against binding energy.

In our study, the decoration of the GO nanoparticles with Cu has been conducted through a simple protocol, and the efficiency of the decoration process has been demonstrated through XPS. The Cu 2p peak position from the survey spectra, along with the Cu 2p high-resolution XPS spectra (Figure 14) are in good agreement with the literature data, which analyzes copper-based materials such as CuO with photoelectrons originating from ground state configuration [34]. Thus, the XPS analysis suggests that the synthesized hybrid nanostructures are decorated with copper oxide, which is in an oxidation state of two through the presence of corresponding Cu 2p_3/2_ and Cu 2p_1/2_ at 935 eV and, respectively, 955 eV. Furthermore, the satellite peaks (marked with arrows) located at 943 eV and 963 eV are also mentioned to derive from CuO [35].

The synergistic effect observed in the combination of Cu and GO nanoparticles is significant because it leads to an increase in the latent heat of the NEPCM (nano-enhanced phase change material) compared to the individual contributions of Cu or GO nanoparticles alone. This effect arises from the enhanced thermal conductivity provided by the Cu nanoparticles and the large surface area and high dispersion capability of the GO nanoparticles, which together facilitate better heat transfer and energy storage within the PCM.

Another aspect that must be considered is to ensure the stability of the Cu@GO nanomaterial over multiple phase change cycles. The concentration of Cu@GO nanoparticles within the phase change material (PCM) is a critical factor in ensuring stability. It should be carefully controlled, as studies suggest that concentrations above 2% by mass may lead to instability, such as sedimentation or agglomeration. These issues could reduce the material’s effectiveness over time. By keeping the concentration below this threshold, a homogeneous distribution can be maintained, and phase separation during repeated thermal cycles can be prevented. Achieving a uniform dispersion of Cu@GO nanoparticles within the PCM is also crucial for stability. Techniques like ultrasonication and the use of surfactants (e.g., Tween 40) play a significant role in this process. They enhance dispersion, thereby improving the material’s stability by minimizing the risk of nanoparticle aggregation and ensuring consistent performance during phase transitions.

Experimental approaches to test stability include micro-computed tomography (micro-CT) to assess the homogeneity of the nanoparticle dispersion after multiple thermal cycles. Additionally, rheological tests can be conducted to evaluate changes in the material’s viscosity and flow behavior, indicative of stability under thermal cycling conditions. These tests can reveal any degradation in the material’s structure or thermal properties over time.

Even though the stability of the newly proposed NEPCM has been investigated through multiple cycles, we will continue to investigate the way this material acts in the long run, after being subjected to phase change cycles during a year. The potential for chemical interactions between the nanoparticles and the base PCM over extended periods must also be investigated. This includes studying any possible reactions that might lead to a reduction in effectiveness or the release of unwanted by-products.

Cu-decorated carbon-based nano species have been previously investigated for the fabrication of supercapacitors [36], as catalysts [37], for coatings [38], and antibacterial materials [39]. But to the best of our knowledge, this is the first work reporting these species for the synthesis of NEPCM. Although the cost of these materials is relatively high, our results show that Cu@GO nanoparticles show potential in the synthesis of NEPCM.

The production of NEPCMs has a moderate environmental footprint, primarily due to energy-intensive processes for the mixing step. However, the enhanced energy storage efficiency of NEPCMs potentially offsets these impacts by reducing overall energy consumption in buildings.

The widespread use of CuO nanoparticles and GO has led to increased human exposure and raised concerns about the potential toxicity risks to human health and the environment [40,41,42,43]. Therefore, the release of these nanoparticles into the environment has garnered significant attention. Research indicates that the toxicity of these materials depends on their dimension, exposure time, ingestion type, and fabrication methodology [43,44,45]. Despite the increasing number of studies in the field, many aspects concerning the ecotoxicology of these nanoparticles should be clarified [46,47]. Still, specific fabrication procedures and particular safety rules must be followed to reduce potential injuries. Future studies should be conducted to investigate the toxicity of the material we discussed here.

## 5. Conclusions

In a period dedicated to diminishing energy consumption and optimizing resource utilization, exhaustive exploration of how to realize these goals is essential. The study introduces a novel nanomaterial, Cu@GO achieved through the decoration of graphene oxide (GO) with copper (Cu), demonstrating a clear increase in latent heat-storage capacities. Compared to individual nanomaterial constituents, this composite mixture demonstrates an increase of over 10% in latent heat capacity.

Based on the experiments, the following findings can be summarized as follows.

From a rheological point of view, the nanoparticles’ loading ratio rather than their type causes a more pronounced response to the compositions’ flow. Most synthesized formulations exhibit non-Newtonian behavior regardless of the loading ratio or testing temperature. However, in some cases, at higher shear rates (above 10 s^−1^), the compositions tend to have a Newtonian behavior.

Of the synthesized compositions, NEPCM obtained following the addition of Cu@GO in loading ratios of 2 and 4% (*w*/*w* nanoparticles with respect to RT31) presents a Newtonian behavior at shear rates above 10 s^−1^ at 30 °C and 70 °C. These findings indicate that adding Cu@GO at these loading ratios does not significantly modify the organic matrix’s flow behavior.

Generally, no important changes could be noted in the case of phase change peak temperature variation.

In the case of the pure material, the solidification phase change temperature is 1.12 °C higher than in the case of melting. A reversal of this trend is also determined by the addition of GO in all of the studied concentration sizes. 

The main finding of the article states that the combination of the two nano species (Cu and GO) leads to an improved thermal response. The decoration of the carbon-based nanoparticles with Cu leads to higher latent heat values for the RT31Cu@GO NEPCM when compared to the RT31Cu and RT31GO, thus proving the synergistic effect of the decorated nanoparticles.

The integration of this newly proposed nanomaterial will pave the way to further improvements in heat storage in buildings, representing a significant step forward in fortifying energy efficiency and sustainable resource utilization across diverse technological domains.

The authors should conduct further research in the next period to investigate the stability of the newly proposed nanomaterial over multiple phase change cycles. While the NEPCMs demonstrated significant improvements in thermal storage capabilities, future work should also investigate the extent and potential environmental risks associated with the use of these advanced materials.

## Figures and Tables

**Table 1:** Formulations’ denomination and nanopowder loading ratio.

Composition	Nanopowder	Paraffin	Mass ratio (Nano/Paraffin) (*w*/*w*)
R31	-	R31	-
R31GO 1	Graphene oxide		1:100
R31GO 2			2:100
R31GO 4			4:100
R31Cu@GO 1	Graphene oxide +1% CuO		1:100
R31Cu@GO 2			2:100
R31Cu@GO 4			4:100
R31Cu 1	CuO		1:100
R31Cu 2			2:100
R31Cu 4			4:100

**Table 2:** Surface elemental composition for the neat and Cu functionalized GO.

Statistics	C1s % at.	O1s % at.	Cu2p % at.	N1s % at.	S2p % at.
GO	93.9	4.9	-	1.2	-
Cu@GO	89.4	7.2	1.2	1.1	1.1

**Table 3:** The thermophysical properties of the studied samples determined with DSC at 1 K/min.

Sample	Process	T_on_ [°C]	T_p_ [°C]	T_end_ [°C]	Q_P_ [W/g]	L [J/g]
RT31	Heating	21.9	30.8	34.4	0.45	138.149
	Cooling	34.8	31.9	21.6	0.30	120.580
RT31Cu 1	Heating	22.4	31.6	35.4	0.28	106.580
	Cooling	33.2	29.8	22.1	0.35	103.223
RT31Cu 2	Heating	21.9	31.1	35.0	0.41	115.057
	Cooling	33.2	32.2	21.6	0.31	112.593
RT31Cu 4	Heating	21.9	31.0	35.0	0.38	103.373
	Cooling	33.2	32.2	21.6	0.30	100.035
RT31GO 1	Heating	21.4	31.2	35.5	0.38	130.852
	Cooling	33.2	30.6	21.6	0.33	124.459
RT31GO 2	Heating	21.9	31.3	35.5	0.39	128.493
	Cooling	33.2	29.8	21.6	0.34	125.135
RT31GO 4	Heating	21.9	31.0	34.5	0.38	126.545
	Cooling	33.2	29.6	21.6	0.35	125.635
RT31Cu@GO 1	Heating	22.2	30.3	33.8	0.45	129.420
	Cooling	32.7	32.4	21.5	0.46	129.330
RT31Cu@GO 2	Heating	21.7	30.2	34.4	0.48	137.610
	Cooling	32.7	32.3	21.5	0.44	134.880
RT31Cu@GO 4	Heating	21.8	30.2	34.3	0.44	135.080
	Cooling	32.7	32.3	21.4	0.44	131.610

## Data Availability

Data will be available on request from the authors.

## References

[1] Ma Z., Lin W., Sohel M.I. (2016). Nano-enhanced phase change materials for improved building performance. Renew. Sustain. Energy Rev..

[2] Sharma A., Chauhan R., Kallioğlu M.A., Chinnasamy V., Singh T. (2021). A review of phase change materials (PCMs) for thermal storage in solar air heating systems. Mater. Today—Proc..

[3] Khan M.M.A., Ibrahim N.I., Mahbubul I.M., Ali H.M., Saidur R., Al-Sulaiman F.A. (2018). Evaluation of solar collector designs with integrated latent heat thermal energy storage: A review. Sol. Energy.

[4] Sadeghi G., Mehrali M., Shahi M., Brem G., Mahmoudi A. (2022). Progress of experimental studies on compact integrated solar collector-storage retrofits adopting phase change materials. Sol. Energy.

[5] Ghalambaz M., Zadeh S.M.H., Mehryan S.A.M., Haghparast A., Zargartalebi H. (2020). Free convection of a suspension containing nano-encapsulated phase change material in a porous cavity; local thermal non-equilibrium model. Heliyon.

[6] Shoeibi S., Kargarsharifabad H., Mirjalily S.A.A., Sadi M., Arabkoohsar A. (2022). A comprehensive review of nano-enhanced phase change materials on solar energy applications. J. Energy Storage.

[7] Elbrashy A., Aboutaleb F., El-Fakharany M., Essa F.A. (2023). Experimental study of solar air heater performance with evacuated tubes connected in series and involving nano-copper oxide/paraffin wax as thermal storage enhancer. Environ. Sci. Pollut. Resear..

[8] Wang Q., Yang L., Song J. (2023). Preparation, thermal conductivity, and applications of nano–enhanced phase change materials (NEPCMs) in solar heat collection: A review. J. Energy Storage.

[9] Khabisi M.A., Roudini G., Barahuie F., Sheybani A., Ibrar M. (2023). Evaluation of phase change material-graphene nanocomposite for thermal regulation enhancement in buildings. Heliyon.

[10] Punniakodi B.M.S., Senthil R. (2022). Recent developments in nano-enhanced phase change materials for solar thermal storage. Sol. Energy Mat. Sol. C..

[11] Mebarek-Oudina F., Chabani I. (2023). Review on nano enhanced PCMs: Insight on nePCM application in thermal management/storage systems. Energies.

[12] Kumar A., Saha S.K., Kumar K.R., Rakshit D. (2022). Study of melting of paraffin dispersed with copper nanoparticles in square cavity subjected to external magnetic field. J. Energy Storage.

[13] Anandan S.S., Sundarababu J., Ravi R., Venkatesan K. (2022). Characteristic study of modified nano CuO powder-based paraffin composite and its experimental investigation of melting/solidification behavior for mobilized cold thermal storage systems. Proc. Inst. Mech. Eng. Part C J. Mech. Eng. Sci..

[14] Venkateshwar K., Joshy N., Simha H., Mahmoud S. (2019). Quantifying the nanoparticles concentration in nano-PCM. J. Nanopart. Res..

[15] Dumitriu C.Ș., Bărbulescu A. (2007). Studies on the Copper Based Alloys Used in Naval Constructions-Modeling the Mass Loss in Different Media.

[16] Bărbulescu A., Dumitriu C.S. (2007). Models of the mass loss of some copper alloys. Chem. Bull. Politeh. Univ..

[17] Dumitriu C.Ș., Bărbulescu A. (2022). Artificial Intelligence Models for the Mass Loss of Copper-Based Alloys under Cavitation. Materials.

[18] Lin S.C., Al-Kayiem H.H. (2016). Evaluation of copper nanoparticles–Paraffin wax compositions for solar thermal energy storage. Sol. Energy.

[19] Zhu D., Wang L., Yu W. (2018). Intriguingly high thermal conductivity increment for CuO nanowires contained nanofluids with low viscosity. Sci. Rep..

[20] Han Z., Fina A. (2011). Thermal conductivity of carbon nanotubes and their polymer nanocomposites: A review. Prog. Polym. Sci..

[21] Muhulet A., Tuncel C., Miculescu F., Pandele A.M., Bobirica C., Orbeci C., Palla-Papavlu A., Voicu S.I. (2020). Synthesis and characterization of polysulfone–TiO 2 decorated MWCNT composite membranes by sonochemical method. Appl. Phys. A.

[22] Olăreț E., Voicu Ș.I., Oprea R., Miculescu F., Butac L., Stancu I.-C., Serafim A. (2022). Nanostructured polyacrylamide hydrogels with improved mechanical properties and antimicrobial behavior. Polymers.

[23] Parsazadeh M., Duan X. (2017). Numerical and statistical study on melting of nanoparticle enhanced phase change material in a shell-and-tube thermal energy storage system. Appl. Therm. Eng..

[24] Chen J., Li L. (2020). Effect of oxidation degree on the thermal properties of graphene oxide. J. Mater. Resear. Technol..

[25] Yang Y., Cao J., Wei N., Meng D., Wang L., Ren G., Yan R., Zhang N. (2019). Thermal conductivity of defective graphene oxide: A molecular dynamic study. Molecules.

[26] Cote L.J., Cruz-Silva R., Huang J. (2009). Flash reduction and patterning of graphite oxide and its polymer composite. J. Am. Chem. Soc..

[27] Hayat M.A., Yang Y., Li L., Bevilaqua M., Chen Y. (2023). Preparation and thermophysical characterisation analysis of potential nano-phase transition materials for thermal energy storage applications. J. Mol. Liq..

[28] Samuel A.G., Subramanian S., Vijendran V., Bhagavathsingh J. (2022). Copper (II)-bis-cyclen intercalated graphene oxide as an efficient two-dimensional nanocomposite material for copper-catalyzed azide-alkyne cycloaddition reaction. Front. Chem..

[29] Yang G., Zhang G., Sheng P., Sun F., Xu W., Zhang D. (2012). A new approach to reduced graphite oxide with tetrathiafulvalene in the presence of metal ions. J. Mater. Chem..

[30] Zhuang Y., Liu Z., Xu W. (2021). Experimental investigation on the non-Newtonian to Newtonian rheology transition of nanoparticles enhanced phase change material during melting. Colloids Surf. A Physicochem. Eng. Asp..

[31] Liu Z., Huang S.-M., Wang C., Zhuang Y. (2023). A review on non-Newtonian effects and structure-activity relationship of nanoparticles enhanced phase change materials in porous media. J. Energy Storage.

[32] Bharathiraja R., Ramkumar T., Selvakumar M. (2023). Studies on the thermal characteristics of nano-enhanced paraffin wax phase change material (PCM) for thermal storage applications. J. Energy Storage.

[33] Elarem R., Alqahtani T., Mellouli S., El Awadi G.A., Algarni S., Kolsi L. (2022). Experimental investigations on thermophysical properties of nano-enhanced phase change materials for thermal energy storage applications. Alex. Eng. J..

[34] Boruban C., Esenturk E.N. (2018). Activated carbon-supported CuO nanoparticles: A hybrid material for carbon dioxide adsorption. J. Nanopart. Res..

[35] Ramachandran T., Pachamuthu M.P., Karthikeyan G., Hamed F., Rezeq M. (2024). Synergistic effects in CuO/SnO_2_/Ti_3_C_2_Tx nanohybrids: Unveiling their potential as supercapacitor cathode material. Mat. Sci. Semicon. Proc..

[36] Kumar B.V.N., Balla P.K., Chirauri S.K., Rao T.K.V., Ramakrishna Y., Rao K.R. (2018). Synthesis and characterization of copper particles decorated reduced graphene oxide nano composites for the application of supercapacitors. AIP Conf. Proc..

[37] Zhang K., Suh J.M., Lee T.H., Cha J.H., Choi J.-W., Jang H.W., Varma R.S., Shokouhimehr M. (2019). Copper oxide–graphene oxide nanocomposite: Efficient catalyst for hydrogenation of nitroaromatics in water. Nano Conv..

[38] Song X., Xie L., Zhang M., Wang W., Li L., Lei P., Liu D., Chen Y., Chen H., Zhao C. (2021). Cu-decorated graphene oxide coatings with enhanced antibacterial activity for surface modification of implant. Mater. Res. Bull..

[39] Shukla A.K., Alam J., Mishra U., Alhoshan M. (2023). Effect of Cu-doped GO nanoparticles on polyphenylsulfone nanocomposite membrane surface and its application for the removal of organic pollutants and antibacterial analysis. Mater. Today Commun..

[40] Ou L., Song B., Liang H., Liu J., Feng X., Deng B., Sun T., Shao L. (2016). Toxicity of graphene-family nanoparticles: A general review of the origins and mechanisms. Part. Fibre Toxicol..

[41] Naz S., Gul A., Zia M., Javed R. (2020). Toxicity of copper oxide nanoparticles: A review study. IET Nanobiotechnol..

[42] Assadian E., Zarei M.H., Gilani A.G., Farshin M., Degampanah H., Pourahmad J. (2018). Toxicity of Copper Oxide (CuO) Nanoparticles on Human Blood Lymphocytes. Biol. Trace Elem. Res..

[43] Ghulam A.N., Dos Santos O.A.L., Hazeem L., Pizzorno Backx B., Bououdina M., Bellucci S. (2022). Graphene Oxide (GO) Materials-Applications and Toxicity on Living Organisms and Environment. J. Funct. Biomater..

[44] Jiang T., Amadei C.A., Lin Y., Gou N., Rahman S.M., Lan J., Vecitis C.D., Gu A.Z. (2021). Dependence of Graphene Oxide (GO) Toxicity on Oxidation Level, Elemental Composition, and Size. Int. J. Mol. Sci..

[45] Yadav S., Singh Raman A.P., Meena H., Goswami A.G., Bhawna K., Kumar V., Jain P., Kumar G., Sagar M., Rana D.K. (2022). An Update on Graphene Oxide: Applications and Toxicity. ACS Omega.

[46] Montagner A., Bosi S., Tenori E., Bidussi M., Alshatwi A.A., Tretiach M., Prato M., Syrgiannis Z. (2017). Ecotoxicological effects of graphene-based materials. 2D Mater..

[47] Ray P.C., Yu H., Fu P.P. (2009). Toxicity and environmental risks of nanomaterials: Challenges and future needs. J. Environ. Sci. Health Part C Environ. Carcinog. Ecotoxicol. Rev..

